# The Inhalation of Sulphuric Ether in Intermittent Fever and Tic Douloureux

**Published:** 1847-11

**Authors:** G. M. Wharton

**Affiliations:** Nashville, Tennessee


Art. V.—*The Inhalation of Sulphuric Ether in Intermittent Fever and Tic Douloureux.* By G. M. Wharton, M.D., of Nashville, Tenn.

Almost every medical journal contains something relative to the effects of the inhalation of sulphuric ether. The entire immunity from pain secured by it in surgical operations has suggested the propriety of its employment in a variety of cases attended with considerable pain. It has produced astonishing results in obstetrical practice, though its indiscriminate use in such contingencies cannot be free from danger. It would doubtless be more frequently resorted to during the progress of certain painful maladies, but that they are generally incurable and eventually fatal, and the constant respiration of ether might establish a habit of intoxication very readily acquired, as has already been reported, which would be more deplorable in its consequences than the original affection.

It is now universally conceded that sulphuric ether, properly prepared and judiciously administered, may be breathed with impunity. As obtained in the shops, it often exists in an impure state, an excess of sulphuric acid and alcohol being present. The untoward symptoms which have sometimes been presented in the experiments of physicians may have been caused by this circumstance. The ether, therefore, should never be prescribed until it has been at least twice washed according to the directions of the dispensatory. Even then there will be found individuals who, owing either to unreasonable alarm or to undefinable idiosyncrasies, cannot inhale the ether without evident embarrassment, the muscles becoming rigid, the pulse small and greatly accelerated, with delirium variously manifested, terminating sometimes in extreme nausea and vomiting, or in syncope, rarely, however, of long continuance. Fortunately persons thus peculiarly affected are few in number, and premonitions are easily remarked which advise the experimenter when to desist; nor, so far as my reading extends, has any credible record been made of death or fatuity following the introduction of ether into the pulmonary apparatus.

The ordinary phenomena which show themselves during etherization are a momentary excitement of the circulation, the number of beats of the heart increasing occasionally from eighty to a hundred and twenty or thirty, perfect relaxation of the muscular system, a mild diaphoresis, in some cases replaced by a hot and dry skin, and an exalted state of the mind, inducing feelings of ecstasy and delight, though now and then patients represent themselves as being visited by a sense of oppression and by dreams and visions of a reverse character.

It occurred to me, and I have not seen the idea intimated elsewhere, that these the most usual phenomena are the opposite of those apparent in the cold stage of intermittent fever; and that if artificially instituted just before the accession of the chill, this might be averted, especially if the sulphate of quinine, the specific in ague, were used in combination with the ether. The impression of the latter might be rendered more permanent, the sedation more soothing and persistent, by adding some extract of opium not incompatible with the other ingredients, as the sulphate of morphia. The formula then might be thus expressed: ℞. Sulph. morphia gr.j, sulph. quinine gr.iv, sulphuric ether (washed) ℥j. M. With the two first articles, singly or united, a chill may be anticipated, yet both frequently fail. Another agent, however, is introduced into this combination which I contend would alone produce the desired result, namely, prevention of the paroxysm. But when administered in conjunction with the sulphates of quinine and morphia, the tonic and sedative entering the circulation at once, their peculiar impressions would be more readily made, their disagreeable taste avoided, a sense of pleasure felt by the patient, and, the ether contributing much to the benefit derived, a cure, I am confident, more certainly effected. The effect of this vaporizing mixture soon subsiding, so far, at least, as the relaxation brought on by the ether is concerned, it will be necessary to renew the inhalation an hour, perhaps, and subsequently fifteen minutes, previous to the time of the expected chill.

There are cases of tic douloureux also, in which periodicity is observed by the disease, and which are relieved by quinine and morphia. In these I feel sure a similar practice would be found effectual.

The subject is interesting and worthy of investigation. Substances have heretofore been introduced into the system by absorption, with a view to remedy diseases, and intermittent fever among the rest. Here, however, the dermoid method was used, though not with any marked success. The lungs offer a much more promising medium than the skin.

Since writing the foregoing, I have been informed of a case in point by Dr. B. F. Hall, an intelligent dentist of this city, who employs the 'letheon' in his profession, and to whom I communicated my views above expressed. A gentleman who had long been troubled with intermittent fever, and had subjected himself in vain to the routine practice in such cases, calling on him a few weeks ago to have a tooth extracted, requested that the operation might be speedily performed, as the time of his chill had almost arrived. He inhaled the ether, and the tooth was extracted as usual without pain. The gentleman escaped the ague which he had confidently expected, and returning some time afterwards, informed the doctor that —he knew not why—he was entirely cured of his chills.

Everything would tend *a priori* to favor the theory I have submitted; and though we should not predicate too much on a solitary case, it should nevertheless, under such circumstances, have no little influence. The ether in the case just narrated may, in addition to the relaxation of the system which it produced, have acted as a mental revulsive—another argument, however, for the employment of the ethereal combination which I have suggested.

Nashville, September 21, 1847.

